# The protective effect of *Azadirachta indica* (neem) against metabolic syndrome: A review

**DOI:** 10.22038/ijbms.2021.48965.11218

**Published:** 2021-03

**Authors:** Fatemeh Yarmohammadi, Soghra Mehri, Nahid Najafi, Sanaz Salar Amoli, Hossein Hosseinzadeh

**Affiliations:** 1 Student Research Committee, Mashhad University of Medical Sciences, Mashhad, Iran; 2 Department of Pharmacodynamics and Toxicology, School of Pharmacy, Mashhad University of Medical Sciences, Mashhad, Iran; 3 Pharmaceutical Research Center, Pharmaceutical Technology Institute, Mashhad University of Medical Sciences, Mashhad, Iran; 4 Department of Clinical Biochemistry, Faculty of Medicine, Mashhad University of Medical Sciences, Mashhad, Iran

**Keywords:** Azadirachta indica, Diabetes, Hyperlipidemia, Hypertension, Metabolic syndrome, Neem, Obesity

## Abstract

Metabolic syndrome is a condition associated with obesity, diabetes, dyslipidemia, and high blood pressure. Recently, the use of phytochemicals is suggested in the control and treatment of metabolic syndrome. The *Azadirachta indica* (neem) is an evergreen tree belonging to the family of Meliaceae. Multiple studies have been confirmed the anti-diabetic and anti-hypertension, anti-hyperlipidemia, and anti-obesity effects of neem. In this review, we reported the protective effects of neem against the complications of metabolic syndrome with a special focus on mechanisms that are involved. It has been shown that neem can control hyperglycemia and hypertension through over-expression of transcription factor nuclear factor erythroid 2–related factor 2 (Nrf2) and anti-oxidant effects. Neem also reduced the glucose uptake through up-regulation of glucose transporter 4 (GLUT4) and inhibition of key intestinal enzymes such as glucosidases. Moreover, neem showed anti-hypertensive effects possibility via the block of calcium channels, up-regulation of endothelial nitric oxide synthase (eNOS), and extracellular signal-regulated kinases 1/2 (ERK1/2) signaling pathway. Anti-oxidant effects play an important role in protective mechanisms of neem against metabolic syndrome and its complications.

## Introduction

Metabolic syndrome (MetS) is a common metabolic disorder that is described for more than multiple decades. The MetS is also known as insulin resistance syndrome and syndrome X (1). Physical inactivity, smoking, increasing age, obesity, and positive family history are risk factors associated with its development (2). Epidemiologic data have been suggested that the prevalence of MetS among the population over 60 years is the highest, and it is increasing among children and adolescents (3). People with MetS have a higher risk of type 2 diabetes and cardiovascular disease (CVD) (4, 5). Also, hypertension and an increase in triglyceride (TG)/high-density lipoprotein (HDL) cholesterol ratio are important components of the MetS and are one of the most risk factors for CVD (6, 7).

Moreover, several studies have been shown other side effects of MetS such as fatty liver disease (8), cirrhosis (9), and polycystic ovary syndrome (10). The pathogenesis of MetS has not been clearly defined, but insulin resistance, oxidative stress, and chronic inflammation are key pathogenic factors of it. Insulin resistance has a role in the development of diabetes mellitus. It has been reported that oxidative stress accelerates the development of complications of MetS. The activation of the inflammatory pathway leads to insulin resistance and diabetes (11, 12).

The first-line treatment of MetS is lifestyle modification on diet, weight, and physical activity. Second-line therapy for patients with MetS is drug therapy (13). In line with the treatment of MetS, the use of herbs has been regarded. Medicinal plants contain bioactive compounds with various metabolic effects. In several studies have been reported the protective potential of plants and herbs against MetS such as *Capsicum* *annuum* L. (14), *Crataegus* *pinnatifida* (15), and green tea (16). Taken together, the management of complications of MetS is the aim of the treatment in these patients, and medicinal plants can play an important role in its treatment.

Neem (*Azadirachta* *indica*) is an evergreen tree of southeastern Asia that is widely distributed in the Indian subcontinent. The height of this tree is approximately 15-20 m and sometimes even up to 35-40 m. The word *A. indica* was derived from the Persian language. The Azad means “free,” and the dirakht is meaning “tree,” and “I” refer to “Indian origin.” (17). Neem is a common name, and also it’s known with the name of Nimbay, Veppai, Ariyaveppu, Vepa in India (18). More than 300 compounds are derived from different parts of neem, such as leaves, flower, seed, fruit, bark, and root. Non-isoprenoids and isoprenoids metabolites are two major groups of these compounds. Some active constituents of neem include nimbidin, sodium nimbidate, nimbin, nimbolide, gallic acid, azadirachtin, and polysaccharides (19). Nimbidin, as a major constituent extracted from neem seeds, demonstrated several biological activities such as anti-inflammatory, anti-pyretic, anti-diabetic, anti-fungal, and anti-ulcer activities. The spermicidal activity of nimbin has been reported in humans. Nimbolide has been shown to exert anti-malaria and antibacterial effects (20). Several studies have been reported the different pharmacologic effects of neem, including hypolipidemic (21), hepatoprotective (22), antimicrobial, anticancer, and anti-diabetes (23) properties. In line with these properties, the US National Academy of Science (NAS) has stated the neem tree as a tree that is solving global problems (24). On the other hand, neem oil has shown vomiting, diarrhea, acidosis, drowsiness, and encephalopathy in human studies. Also, mild to severe changes in the liver, intestine, spleen, kidney, and heart of chick and genotoxicity and anti-fertility in mice and rats by neem leaves and seeds have been reported. Neem leaf extract also decreased sperm count and sperm motility, probably due to androgen deficiency. Nimbolide has induced the kidney, small intestine, liver dysfunction, and blood pressure drop suddenly in animals (20). This review focuses on the effects of neem in treatment of diabetes, high blood pressure, dyslipidemia, and obesity. 

***Methodology***

The databases of PubMed, Scopus, and Google Scholar have been involved in this review. Articles have been collected from the date of inception up to January 2020. The search keywords included metabolic syndrome, hypertension, blood pressure, hypotensive, antihypertensive, dyslipidemia, hyperlipidemia, high cholesterol, high triglyceride, hypercholesterolemia, hypertriglyceridemia, atherogenic, atherosclerosis, obesity, overweight, appetite, anti*-*obesity, weight loss, diabetes, hyperglycemia, insulin, hypoglycemic, antihyperglycemic, antidiabetic, blood glucose, neem, and *Azadirachta* *indica*.

***Effects of neem on metabolic syndrome***

*Effects of neem on high blood pressure*

**Figure 1:**
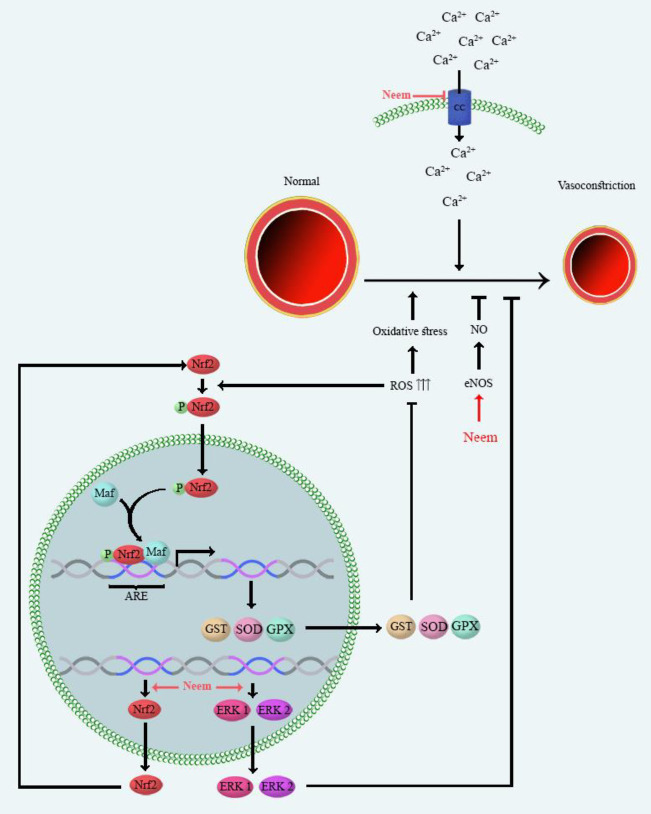
Main mechanisms of neem on high blood pressure. Mechanisms underlying the protective effect of neem against high BP are including the block of calcium channels, up-regulation of ERK 1/2 and Nrf2 gene expression, and normalize serum of NO bioavailability. ARE: Anti-oxidant response element; eNOS: endothelial nitric oxide synthase; ERKs: extracellular-regulated kinases; GPX: glutathione peroxidase; GST: glutathione S-transferases, Nrf2: nuclear factor erythroid-2-related factor 2; NO: nitric oxide; ROS: reactive oxygen species; SOD: superoxide dismutase

One of the main constituents of MetS is high blood pressure (BP). Effects of several plants investigated on BP such as *Aloe vera* (25) and *Capsicum annuum* L. (14). High BP has an increased risk of heart and blood vessel diseases. Multiple mechanisms induce high BP, including: (1) Calcium channels initiate vascular smooth muscle contraction through the release of calcium, which is mediated by Ca^2+ ^influx via L-type and voltage-gated calcium channels. Calcium channels have an important role in the induction of high BP (26); (2) the extracellular signal-regulated kinase (ERK 1 and 2) are from the mitogen-activated protein kinases (MAPK) family. ERK1 and ERK2 play an essential role in the regulation of vascular smooth muscle contraction. Down-regulation of ERK 1 and 2 genes reduces both vascular smooth muscle cell growth and vasoconstriction. Therefore, ERK 1and 2 are a target for the induction of high BP (27); (3) Nitric oxide (NO) is a vasodilator produced by nitric oxide synthase (NOS) enzymes. The NOS isozymes and NO level are candidates for involvement in high BP (28); (4) Nuclear factor erythroid 2–related factor 2 (Nrf2) as a transcription factor involves transcriptional induction of several anti-oxidant genes. Nrf2 regulates signaling pathway functions to reduce reactive oxygen radicals (ROS) production. The down-regulation of Nrf2 expressions induces ROS production and resulting in high BP. The contraction of smooth muscle and depletion of NO are the mechanisms of ROS-induced high BP (29). Several studies have been reported the beneficial effects of different extracts (aqueous, alcoholic) of neem leaves against high BP which have been categorized in Table 1. The mechanisms underlying the protective effect of neem against high BP have been presented in Figure 1. The mechanisms are including the block of calcium channels (30), up-regulation of ERK 1 and 2 (31) and Nrf2 gene expression, reduction of oxidative stress markers, and elevation of the nitric oxide (NO) levels (32). It has been reported that neem exerted the vasodilatation effects possibility through the block of calcium channel in the isolated aorta of rat and rabbit. Also, it has been shown that neem exerted dose-dependent fall in arterial pressure of isolated guinea-pig atrial (30). The down-regulation of ERK1 and 2 have been reported in cardiac and renal tissues of rats treated by sodium fluoride (600 ppm in drinking water). Neem protected hypertensive rats through (100 and 200 mg/kg, p.o.) up-regulation of ERK (31). L-NAME (N ω -Nitro-L-Arginine Methyl Ester) is a NOS inhibitor and reduces NO bioavailability. Polyphenol-rich fraction of neem (100 and 200 mg/kg) restored NO level in rats were treated with L-NAME (orally, 40 mg/kg) (32). The methanol extract of neem (orally, 100 and 200 mg/kg for 7 days) increased NO level in serum of rats exposed to sodium fluoride (NaF) (600 ppm in drinking water) (31). The crude (0.3-3 mg/ml), aqueous (1-5 mg/ml) and ethyl acetate (0.1-1 mg/kg) extracts of neem induced endothelium-dependent vasorelaxation in isolated rat aorta (30). 

Neem restored anti-oxidant enzyme activity, including superoxide dismutase (SOD), glutathione peroxidase (GPx), and glutathione S-transferase (GSTs) in animal models of high BP. Neem also improved glutathione (GSH) and reduced malondialdehyde (MDA) and protein carbonyl (markers of oxidative stress) levels (31, 32). The aqueous extract of neem (20 mg/kg) reduced mean arterial pressure in rats which were treated by DOCA-salt (15 mg/kg, s.c.) and drinking water containing 1.0% NaCl and 0.03% KCl (33).

A study has been reported on the effect of neem leaves on high BP of the 90 diabetic patients aged 40-60, which were kept under observation for a month. During the study, patients received 2 g powder of neem daily for three months. A significant reduction was observed in the BP of treated patients (34).

*Effects of neem on hyperlipidemia*

One of the most components of MetS is hyperlipidemia. Many medicinal plants showed positive effects on hyperlipidemia such as barberry (*Berberis vulgaris*) (35) and rosemary (*Rosmarinus officinalis*) (36). Plasma lipid levels elevate in people with diabetes and obesity (37). Hyperlipidemia contributes to impair endothelial function, development of atherosclerosis, and coronary heart disease (CHD) through the enhancement of oxidative stress (38). Anti-oxidant defense system (SOD and GPx) protects plasma lipoproteins against oxidative stress (39). The elevation of ROS generation under stress conditions (diabetes and obesity) causes oxidative damage of lipoproteins in the plasma (40, 41). The oxidation of lipoproteins increases TG, very-low-density lipoprotein (VLDL), low-density lipoprotein (LDL) concentrations in plasma (40). The measure of serum TG, total cholesterol (TC), LDL, HDL, and cholesterol is the reference procedure for the determination of lipid profile (42). As mentioned in Table 2 several studies have been shown the effects of neem in the management of hyperlipidemia. 

**Figure 2:**
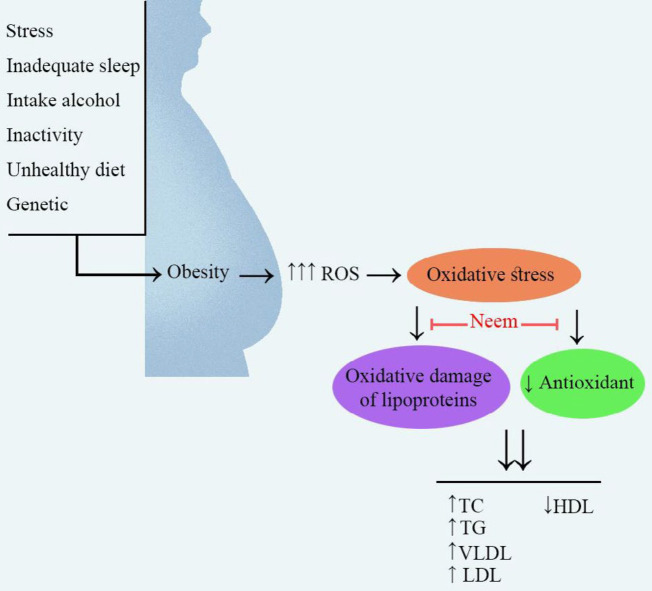
Main mechanisms of neem on hyperlipidemia. Neem has been shown protective effects against hyperlipidemia via improvement of the function of anti-oxidant markers and inhibition of oxidative damage of lipoproteins

HDL: high-density lipoprotein; LDL: low-density lipoprotein; ROS: reactive oxygen species; TC: total cholesterol; TG: triglyceride; VLDL: very-low-density lipoprotein

Different doses of neem (100, 200, 250, 300, 400, and 500 mg/kg) in streptozotocin (STZ)-diabetic rats decreased serum TC, TG, LDL, VLDL levels and increased serum HDL levels (43-48). Also, two different doses of neem (7.5 and 20 mg/kg) in STZ-diabetic mice normalized lipid profile (49, 50). Neem (200, 250, 400, and 500 mg/kg, p.o.) reduced TC, TG, HDL, LDL, VLDL in rats which were treated with alloxan (120 mg/kg, i.p.) (51-53). Also, neem attenuated hyperglycemia, and hyperlipidemia via induction of SOD, catalase (CAT) levels in diabetic rats (46). The aqueous leaf extract of neem (250, 500, and 1000 mg/kg, p.o.) decreased TC and TG levels and increased HDL levels in rats which were treated with isoprenaline (54). The leaf extract of neem (50 and 300 mg/kg/day orally) prevented the rise of TC, LDL, and TG in cholesterol-fed rats (55). The mechanisms which are important in the effects of neem against hyperlipidemia have been shown in Figure 2.

*Effects of neem on obesity*

Obesity and overweight are a serious health problem that is increasing worldwide. Obesity is associated with a life expectancy decrease and a significant increase in mortality (56). Stress, inadequate sleep, intake of alcohol, inactivity, unhealthy diet, age, genetic are some of the risk factors for obesity Figure 2 (57). Diabetes, heart disease, high blood pressure, hyperlipidemia, and atherosclerosis are obesity-related complications (58). Lipase and α-glucosidase are two types of obesity agents that use of their inhibitors can be the ideal therapy for obesity control (59, 60).

The aqueous and methanolic extract of stem bark and roots of neem (520 µg/ml) inhibited pancreatic lipase and α- glucosidase in an *in vitro* system (59). But, the leaf extract of neem as a medicinal plant (500 mg/kg, orally) is not decreased the body weight in rats were treated for 28 days (60). There are a few studies available on the protective effects of neem against obesity which has been included in Table 3. Therefore, the effects of neem on obesity cannot be appropriately explained, and further studies are needed.

*Effects of neem on diabetes*

Diabetes, as a growing public health problem, is characterized by impairment of systemic insulin secretion, reduction of insulin action, and resulting in hyperglycemia (61). Diabetes-associated main complications are nerve damage (62), myocardial infarction (63), atherosclerosis (64), renal failure (65), blindness (66), and limb amputation (67). The microvascular disease has been known as the foremost cause of these complications (68). Glucose-mediated vascular damage occurs as a result of the overproduction of ROS and oxidative stress (69). Enzymatic and nonenzymatic anti-oxidants are defense mechanisms against oxidative stress. Common enzymatic anti-oxidants include SOD, CAT, GPx, and glutaredoxin (GRx). Vitamins A, C, E, and glutathione are common nonenzymatic anti-oxidants (70). Pancreatic beta cells are more susceptible to oxidative stress than other cells because they have relatively low levels of anti-oxidants (71). Therefore, the reduction of ROS and induction of anti-oxidant activity are therapeutic approaches to decrease hyperglycemia and diabetes (72). On the other hand, salivary α-amylase and intestinal glucosidases play an essential role in the digestion of starch to produce glucose in the small intestine (73). Also, the inhibition of these enzymes could be effective in the control of diabetes (74). Several reports have been shown that medicinal plants useful for the management and treatment of diabetes. Some of these plants include* Vernonia amygdalina (*75*)*, *Nigella* *sativa* L. (76), grapes (*Vitis vinifera*) (77), and* Allium sativum *(garlic) (78) which are useful in the remedy of diabetes. The use of the neem is most popular to control diabetes in different regions of the world, such as India (79), Pakistan (80), Bengal (81), Indonesia (82), and Northwest Nigeria (83). Glucagon-like peptide-1 (GLP-1) is a hormone that plays an essential role in the release of insulin and is inactivated by dipeptidyl peptidase IV (DPP-IV) (84, 85). Inhibition of DPP-IV (a peptidase) is a method for diabetes treatment (86). In this method, substrate Gly-Pro-p-Nitroanilide (GPPN) was cleaved to paranitroanilide (a yellow-colored product) by DPP-IV and the absorbance was measured at 380 nm. Inhibitory activity of neem leaves (35 μl with varying concentrations) was determined on DPP-IV activity via this method, and neem exhibited a weak inhibitory activity (17%) on DPP-IV (82).

*• Effects of neem in diabetic human*

Neem is available as a dietary supplement in an herbal mixture in North America. Treatment with this dietary supplement (2 capsules 3 times per day) for 3 months in type 2 diabetic patients (the ages of 18 and 70) improved glucose control and HbA1c levels (87). The study of Kochhar has been investigated the antidiabetic effect of neem in 90 diabetic men 40 to 60 years of age. Subjects received 2 g of neem leaf powder daily for three months. The results of this study showed that neem reduces sweating, headache, burning feet, itching, polydipsia, and polyphagia in diabetic humans (34).

*• Effects of neem in alloxan/streptozotocin-induced diabetic animals*

*•• Effects of neem in alloxan-induced diabetic rats*

Alloxan is a toxic glucose analog that accumulates in pancreatic beta cells via glucose transporter 2 (GLUT2) and inhibits its function. The intraperitoneal injection of alloxan (at doses of 100, 120, and 150 mg/kg) is a conventional method for the induction of diabetes in rat models (88). The oral administration of ethanolic extract of neem in different doses (100 to 800 mg/kg for 14 or 28 days) reduced blood glucose levels in rats which were treated with alloxan (52, 89, 90). The combination of neem (50 mg/kg) with *Gynura procumbens* ethanolic (112.5 mg/kg) extracts (2 times a day for 15 days) increased insulin expression, decreased blood glucose concentration, and improved the morphology of the islets of Langerhans and beta-cells in rats (91). The aqueous extract of neem leaf and bark was effective in reducing oxidative stress markers and lipid peroxidation of the blood sample, liver, and kidney tissues in diabetic rats (92, 93). Polyherbal formulation (PHF) is containing more than one herb that is used all around the world to treat diseases (94). PHFs used in the treatment of diabetes are including Karnim Plus and DIA7. The antidiabetic activity of Karnim Plus and DIA7 is investigated in rats treated with alloxan. Karnim Plus and DIA7 contain neem extract and decrease blood glucose levels in diabetic rats (51, 95). In Table 4, different studies on the effect of neem on diabetes have been summarized. 

*•• Effects of neem in alloxan-induced diabetic rabbits*

The hypoglycemic effect of neem (ethanolic extract of leaves, 200 mg/kg) was observed in rabbits that were treated with alloxan (150 mg/kg, i.v.) (96). Also, leaf extract (500 mg/kg, p.o. daily for six weeks) and seed oil (5 mg/kg, p.o. daily for six weeks) of neem decreased blood glucose in diabetic rabbits (alloxan in a single dose, 140 mg/kg, i.v.) (97).

*•• Effects of neem in streptozotocin-induced diabetic rats*

**Figure 3:**
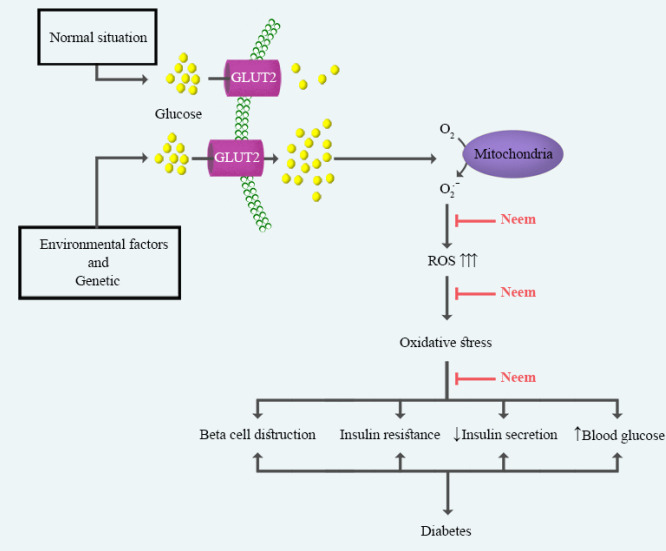
Main mechanisms of neem on diabetes. Neem has been shown protective effects against diabetes via inhibition of the mitochondrial/oxidative stress pathways

GLUT2: glucose transporter 2; ROS: reactive oxygen species

Streptozotocin (STZ) is one of the most diabetogenic agents using in diabetes research. Its mechanisms for the induction of diabetes are inhibition of insulin secretion and the death of the beta-cells (88). In rat models, the injection of STZ (at doses of 35, 45, 55, 60, 65, 70, and 100 mg/kg) is a standard method for the induction of diabetes (47, 48, 98-102). The ethanolic extract of neem leaves (at doses of 200 and 500 mg/kg, p.o.) reduced blood glucose levels in rats were treated with STZ (44). Moreover, the oral administration of neem (leaf ethanolic extract) induced markers of the anti-oxidant system (SOD, CAT, GPx, and GSH levels) and reduced lipid peroxidation in diabetic rats (103) (Figure 3). The aqueous extract of neem leaves (at doses of 100, 200, 250, 400, 500 and 600 mg/kg, p.o.) decreased blood glucose levels and improved serum insulin levels in rats were treated with STZ (45, 47, 100, 101, 104, 105). Moreover, neem (400 mg/kg, p.o. for 30 days) increased insulin receptor protein expression in diabetic rats (STZ: 35 mg/kg, i.p.). It also up-regulated cytosolic and plasma membrane glucose transporter 4 (GLUT4) in the gastrocnemius muscle of diabetic rats (47). Insulin enhances glucose uptake into muscle tissues through GLUT4, and therefore it controls glucose homeostasis (106). Several studies investigated the effects of PHFs on diabetes in rats which were treated with STZ. Allopolyherbal (neem at the dose of 500 mg/kg) (107), Glucova Active (43), Dihar (10 % of neem) (108), MAC-ST/001 (20 g/100 g of neem) (109), and Herbo-mineral (25 mg of neem) (109) are PHFs that decrease blood glucose levels and increase insulin level in STZ-diabetic rat*.*

*•• Effects of neem in streptozotocin-induced diabetic mice*

The decrease of serum glucose levels and increase of glycogen content, plasma insulin, and c-peptide levels with aqueous extract of neem have been shown in mice treated with STZ (110, 111). Also, the increase of glucose-6-phosphate dehydrogenase (G6PD) activity with neem has been shown in diabetic mice (110). The chloroform extract of neem in addition to reducing glucose level and induction of insulin level decreased oxidative stress markers and LPO in mice treated with STZ-nicotinamide. Also, it reduced glucose-6-phosphatase-α (G6Pase), glucokinase (GK), α-amylase, and α-glucosidase activities, and induced HK activity (50). Dianex, an herbal formulation is containing neem at a dose of 7.5 mg/kg. Dianex has been shown hypoglycemic effects in STZ-diabetic mice (49).

*• Effects of neem in glucose-induced diabetic animals*

The aqueous and ethanolic extracts of neem leaves decreased glucose level and increased insulin secretion in rats were treated with glucose (3 mg/ml, p.o.) (112, 113). Also, the potential use of ethanolic extract rhizome of neem (300 mg/kg, p.o.) was investigated in mice that were treated with glucose (1 g/kg). In this study, neem reduced blood glucose (114).

**Table 1:** Effects of neem against high blood pressure

Part (s) of the plant used/ Extract(s)	Neem dose/ route	Study design	Results	Ref
Leaves/ methanolic	100 and 200 (mg/kg), p.o.	Male rats, L-NAM (40 mg/kg), p.o.	↓ BP ↑ NO level ↓Oxidative stress ↑Expressions of Nrf2	(32, 115)
Leaves/ methanolic	100 and 200(mg/kg), p.o.	Male rats, NaF 600 (ppm)	↓ BP ↓Oxidative stress ↑ Expressions of ERK	(31, 116)
Crude/ aqueous	1, 3, 10 and 30 (mg/kg), p.o.	Male and female rats, arterial cannula	↓ BP Blockade Ca^++^ channel ↑ NO	(30)
Crude/ aqueous and ethylacetate	0.01- 10 mg/ml, p.o.	Rabbit, isolated rabbit aorta	↓ BP Blockade Ca^++^ channel ↑ NO	(30)
Crude/ aqueous and ethylacetate	0.001- 10 mg/ml, p.o.	Rats, isolated rat aorta	↓ BP Blockade Ca^++^ channel ↑ NO	(30)
Crude/ aqueous and ethylacetate	0.01- 10 mg/ml, p.o.	Guinea pig, isolated guinea pig atrial	↓ BP Blockade Ca^++^ channel ↑ NO	(30)
Leaves/ aqueous	20 (mg/kg), p.o.	Male rats, DOCA-salt 15 (mg/kg), s.c.	↓ MAP ↓ Alterations of ECG	(33)
Leaves/ alcoholic	100, 300 and 1000, (mg/kg), i.v.	Male rats, atropine (1mg/kg) and mepyramine (3 mg/kg), i.v.	↓ BP	(117)
Leaves	5, 10, 20, 40, 80, 100, and 200, (mg/kg), i.v.	Rabbit and guinea pig, ouabain-induced cardiac dysrhythmias	↓ BP	(118)
Leaves/ aqueous	2 g, p.o.	Male patients (40-60 years)	↓ BP	(34)

↑: increase; ↓: decrease; BP: blood pressure; DOCA: deoxycorticosterone acetate; ECG: electrocardiogram; ERK: extracellular signal-regulated kinase; g: gram; i.v.: intravenous; kg: kilogram; L-NAM: N ω -nitro-L-arginine methyl ester; MAP: mitogen-activated protein; mg: milligram; NO: nitric oxide; Nrf2: Nuclear factor erythroid 2–related factor 2; p.o.: per os (orally); ppm: parts per million; s.c.: subcutaneous

**Table 2:** Effects of neem against hyperlipidemia

**Part (s) of the plant used/ Extract(s)**	**Neem dose/ route**	**Study design**	**Results**	**Ref**
Leaves/ aqueous	250 mg/kg, p.o.	Male and female rats, STZ (60 mg/kg), i.p.	↓ LDL, TG, and cholesterol ↑ HDL	(45)
Leaves/ aqueous	400 mg/kg, p.o.	Male rats, STZ (35 mg/kg), i.p.	Normalized lipid profile	(47)
Leaves/ aqueous	500 mg/kg, p.o.	Rats, STZ (45 mg/kg), i.p.	↓ Cholesterol, TG	(48)
Leaves/ chloroform	300 mg/kg, p.o.	Male rats, STZ (60 mg/kg), i.p.	↓ LPO, ↑SOD, CAT activity, ↑ GSH levels, ↓ GSSG levels	(46)
Leaves/ alcoholic	200 mg/kg, p.o.	Male rats, STZ (50 mg/kg), i.v.	↓TC, ↓ TG, LDL and VLDL	(44)
Seeds/ petroleum ether	0.09 and 2 mg/kg, p.o.	Male rats, STZ (55 mg/kg), i.v.	↓TC, TG	(119)
Allopolyherbal	500 mg/kg, p.o.	Male and female rats, STZ (60 mg/kg), i.p.	↓ TC, TG, LDL, VLDL, serum creatinine, SGOT, and SGPT ↑ HDL	(107)
Glucova Active	_	Rats, STZ (35 and 50 mg/kg), i.p.	↓ Serum cholesterol, TG, LDL, VLDL ↑ HDL	(43)
Dihar	10%, p.o.	Male rats, STZ (45 mg/kg), i.v.	↓ Cholesterol, TG, LDL, Creatinine, Urea and LPO ↑ HDL, SOD and CAT	(108)
Leaves/ chloroform	20 mg/kg, p.o.	Male mice, STZ (60 mg/kg), i.v.	↓ TG, TC, ↑ HDL ↓ LPO	(50)
Dianex	7.5 mg/kg, p.o.	Male and female mice, STZ (60 mg/kg), i.p.	↓ TG, cholesterol, urea and cratininine	(49)
Leaves/ methanolic	500 mg/kg, p.o.	Male and female rats, alloxan (100 mg/kg), i.p.	↑ HDL ↓ LDL and TG	(53)
Leaves/ ethanolic	100 and 250 mg/kg, p.o.	Male rats, alloxan (120 mg/kg), i.p.	↓ TC, TG, HDL, LDL, VLDL	(52)
Leaves/ ethanolic	100 mg/kg, p.o.	Male rats, alloxan (120 mg/kg), i.p.	↓ Serum cholesterol, TG, LDL, creatinine, and urea ↑ HDL	(89)
Karnim Plus	200 and 400 mg/kg, p.o.	Rats, alloxan (120 mg/kg), i.p.	↓ Serum cholesterol, TG, creatinine, and urea	(51)
Ethanolic (leaves)	50 and 300 mg/kg, p.o.	Male rats, cholesterol	↓TC, LDL and TG	(55)
Aqueous (leaves)	250, 500 and 1000 mg/kg, p.o.	Male rats, isoprenaline (25 mg/kg), s.c.	↓TC and TG ↑ HDL	(54)

↑: increase; ↓: decrease; CAT: catalase; GSH: glutathione; GSSG: glutathione disulfide; HDL: high-density lipoprotein; i.p.: intraperitoneal; i.v.: intravenous, kg: kilogram; LDL: low-density lipoprotein; LPO: lipid peroxidation; mg: milligram; p.o.: per os (orally); SGOT: serum glutamic oxaloacetic transaminase; SGPT: serum glutamic pyruvic transaminase, SOD: superoxide dismutase; STZ: streptozotocin; TC: total cholesterol; TG: triglyceride; VLDL: very-low-density lipoprotein

**Table 3:** Effects of neem against obesity

**Part (s) of the plant used/ Extract(s)**	**Neem dose/ route**	**Study design**	**Results**	**Ref**
Leaves/ aqueous	500 mg/kg, p.o.	Male and female rats	No effect	(60)
Stem-bark and roots/ aqueous and methanolic	IC50: 520 µg/ml	*In vitro*	Inhibited pancreatic lipase and α- glucosidase	(59)

IC50: inhibitory concentration; kg: kilogram; µg: microgram; mg: milligram; ml: milliliter; p.o.: per os (orally)

**Table 4:** Effects of neem against diabetes

**Part (s) of the plant used/ Extract (s)**	**Neem dose/ route**	**Study design**	**Results**	**Ref**
Bark root/ ethanolic	200, 400, 800 mg/kg, p.o.	Rats, alloxan (100 mg/kg), i.p.	↓BG	(90)
Leaves and seeds/ ethanolic	500 mg/kg, p.o.	Males rats, alloxan (120 mg/kg), i.p.	↓BG	(120)
Leaves/ ethanolic	200 mg/kg, p.o.	Rats, alloxan (150 mg/kg), i.p.	↓BG	(121)
Leaves/ ethanolic	100 and 250 mg/kg, p.o.	Males rats, alloxan (120 mg/kg), i.p.	↓BG	(52)
Leaves/ ethanolic	250 mg/kg, p.o.	Males rats, alloxan (100 mg/kg), i.p.	↓BG	(122)
Leaves/ ethanolic	100 mg/kg, p.o.	Males rats, alloxan (120 mg/kg), i.p.	↓BG	(89)
Leaves/ ethanolic	200 mg/kg, p.o.	Males rats, alloxan (150 mg/kg), i.p.	↓BG, ↑Insulin Protected beta cells and islets Langerhans	(91)
Leaves and bark/ aqueous	100 and 500 mg/kg, p.o.	Rats, alloxan (150 mg/kg), s.c.	↓BG ↓ Oxidative stress markers and LPO and DNA fragmentation and PKC beta II	(92)
Leaves and bark/ aqueous	100 and 500 mg/kg, p.o.	Rats, alloxan	↓BG ↓ Oxidative stress markers and LPO	(93)
Leaves and bark/ aqueous	75 mg/kg, p.o.	Rats, alloxan (150 mg/kg), i.p.	↓BG	(123)
Leaves/ aqueous	25, 50 and 100 mg/kg, p.o.	Rats, alloxan (150 mg/kg), i.p.	↓BG ↓ Oxidative stress markers and LPO	(124)
Leaves/ aqueous	400 mg/kg, p.o.	Males and females rats, alloxan (150 mg/kg), i.p.	↓BG Improved liver function	(125)
Seeds/ aqueous	500 mg/kg, p.o.	Males and females rats, alloxan (150 mg/kg), i.p.	↓BG	(126)
Leaves/ polyherbal	200 and 400 mg/kg, p.o.	Rats, alloxan (120 mg/kg), i.p.	↓BG	(51)
Leaves/ polyherbal	14.28%	Rats, alloxan (150 mg/kg), i.p.	↓BG	(95)
Leaves/ ethanolic	200 g, p.o.	Rabbits, alloxan (150 mg/kg), i.v.	↓BG	(96)
Leaves/ aqueous	500 ml/kg, p.o.	Males and female rabbits, alloxan	↓BG	(97)
Seeds/ aqueous	5 mg/kg, p.o.	Males and females rabbits, alloxan	↓BG	(97)
Leaves/ chloroform	200 mg/kg, p.o.	Males rats, STZ (50 mg/kg), i.p.	↓BG, ↓ LPO ↑ Antioxidant markers	(104)
Leaves/ chloroform	300 mg/kg, p.o.	Males rats, STZ (65 mg/kg), i.v.	↓BG, ↑ Insulin ↑ Antioxidant markers ↓ LPO	(46)
Leaves/ aqueous	600 mg/kg, p.o.	Males rats, STZ (60 mg/kg), i.p.	↓BG, ↓ LPO ↑ Antioxidant markers protected beta cells and islets langerhans ↑ Pain threshold	(99)
Leaves/ aqueous	600 mg/kg, p.o.	Males rats, STZ (60 mg/kg), i.p.	↓BG, ↓ LPO ↑ Antioxidant markers protected beta cells and islets langerhans	(105)
Leaves/ aqueous	400 mg/kg, p.o.	Males rat, STZ (35 mg/kg), i.p.	↓BG, ↑ Insulin Normalized GluT4	(47)
Leaves/ aqueous	500 mg/kg, p.o.	Rats, STZ (45 mg/kg), i.p.	↓BG, ↓ LPO ↑ Antioxidant markers protected beta cells and islets langerhans	(48)
Leaves/ aqueous	500 mg/kg, p.o.	Males and females rat, STZ (55 mg/kg), i.p.	↓BG, ↓ LPO ↑ Antioxidant markers	(98)
Leaves/ aqueous	250 mg/kg, p.o.	Males rat, STZ (60 mg/kg), i.p.	↓BG	(45)
Leaves/ aqueous	100 mg/kg, p.o.	Males rat, STZ (65 mg/kg), i.p.	↓BG	(100)
Leaves/ aqueous	10 ml/kg, p.o.	Males rat, STZ (65 mg/kg), i.p.	↓BG, ↓ LPO ↑ Antioxidant markers	(127)
Leaves/ aqueous	50, 100, 200 and 400 mg/kg, p.o.	Males and females rat, STZ (50 mg/kg), i.p.	↓BG ↑ Antioxidant markers ↓ LPO	(128)
Leaves/ ethanolic	200 mg/kg, p.o.	Males rat, STZ (65 mg/kg), i.p.	↓BG	(103)
Leaves/ ethanolic	200 mg/kg, p.o.	Males rat, STZ (50 mg/kg), i.v.	↓BG, ↓ LPO ↑ Antioxidant markers	(44)
Leaves/ ethanolic	500 mg/kg, p.o.	Males and females rat, STZ (70 mg/kg), i.p.	↓BG, ↓ LPO ↑ Antioxidant markers protected beta cells and islets langerhans	(101)
Leaves/ ethanolic	500 mg/kg, p.o.	Males rat, STZ (70 mg/kg), i.v.	↓BG, ↓ LPO ↑ Antioxidant markers	(129)
Leaves/ ethanolic	500 mg/kg, p.o.	Males rat, STZ (70 mg/kg), i.p.	↓BG protected beta cells and islets langerhans	(130)
Seeds / ethanolic	1.2 ml, p.o.	Females rat, STZ (100 mg/kg), s.c.	↓BG, ↓ LPO ↑ Antioxidant markers	(102)
Leaves/ ethanolic	200 mg/kg, p.o.	Males rat, STZ (65 mg/kg), i.p.	↓BG, ↓ LPO ↑ Antioxidant markers	(103)
Leaves/ ethanolic	500 mg/kg, p.o.	Males and females rat, STZ (70 mg/kg), i.p.	↓BG	(128)
Seeds/ petroleum ether	0.9 and 2 mg/kg, p.o.	Males rat, STZ (55 mg/kg), i.p.	↓BG, ↓ LPO ↑ Antioxidant markers	(131)
Allopolyherbal	500 mg/kg, p.o.	Males and females rat, STZ (60 mg/kg), i.p.	↓BG, ↑ Insulin	(107)
Glucova Active	_	Rat, STZ (35 and 50 mg/kg), i.p.	↓BG, ↑ Insulin protected beta cells	(43)
Dihar	10%, p.o.	Males rat, STZ (45 mg/kg), i.v.	↓BG, ↑ Insulin protected beta cells	(108)
MAC-ST/001	20 g/100 g, p.o.	Males and females rat, STZ (55 mg/kg), i.p.	↓BG, ↑ Insulin protected beta cells ↓ G6Pase	(109)
Herbo-mineral	25 mg/kg, p.o.	Males rat, STZ (60 mg/kg), i.p.	↓BG, ↑ Insulin	(132)
_	100 g, p.o.	Females rat, STZ (65 mg/kg), i.p.	↓BG, ↑ Insulin protected beta cells ↓ G6Pase	(133)
Leaves/ aqueous	100 μg/ 200 μL, p.o.	Mice, STZ (3 mg/25 g), i.p.	↓BG, ↓ G6Pase	(110)
Seeds/ aqueous	1mg/ml, p.o.	Females mice, STZ (100 mg/kg), s.c.	↓BG	(102)
Leaves and seeds/ aqueous	100, 200, 300 µl, p.o.	Males mice, STZ (55 mg/kg), i.p.	↓BG ↑ Antioxidant markers	(111)
Leaves/ chloroform	20 and 30 mg/kg, p.o.	Males mice, STZ (60-120 mg/kg), i.p.	↓BG, ↑ Insulin ↑ Antioxidant markers ↓ LPO, G6Pase, GK, α-amylase and α-glucosidase activity ↑ HK activity	(50)
Dianex	7.5 mg/kg, p.o.	Males and females mice, STZ (60 mg/kg), i.p.	↓BG	(49)
Leaves/ aqueous	200 mg/kg, p.o.	Males rabbit, STZ (50 mg/kg), i.p.	↓BG, ↑Insulin	(113)
Leaves/ aqueous	400 mg/kg, p.o.	Males and females rat, glucose (3 g/kg), p.o.	↓BG	(112)
Leaves/ ethanolic	_	Rat, glucose (3 mg/ml), p.o.	↓BG	(113)
Rhizome / ethanolic	300 mg/kg, p.o.	Males mice, glucose (1 g/kg), p.o.	↓BG	(114)
Leaves/ aqueous	10 mg/kg, p.o.	Males and females rat	↓BG	(134)
Seeds, stems, flowers, and bark/ aqueous	0.1, 0.092, 0.084, 0.071 and 0.05 g/ml, p.o.	Males rat	↓BG	(135)
Stem bark/ ethanolic	15, 30, 60, 120 and 240 (µg/ml), p.o.	Males rat	↑ Antioxidant markers ↓ LPO	(136)
Plant/ aqueous	25–1000 µg/ml	INS-1 b-cells, glucose (5.6 mM) g/kg	↑ Insulin release ↑ Glucose consumption	(137)
Plant/ aqueous	25–1000 µg/ml	3T3-L1 adipocytes, glucose 5.6 mM) g/kg	↑ Insulin release ↑ Glucose consumption	(137)

↑: increase; ↓: decrease; BG: blood glucose; GK: glucokinase; G6Pase: glucose-6-phosphatase; GluT4: glucose transporter 4; g: gram; HK: hexokinases; INS-1 b-cells: insulin-secreting cells; i.p.: intraperitoneal; I.V.: intravenous; kg: kilogram; LPO: lipid peroxidation; µg: microgram; µl: microliter; mg: milligram; ml:milliliter; PKC: protein kinase C; p.o.: per os (orally); s.c.: subcutaneous; STZ: streptozotocin

## Conclusion

In summary, *A. indica* (neem) is effective in MetS and anti-oxidant effects appear to play an important role in protective mechanisms of neem against MetS and the complications associated with it. Neem increases the expression of Nrf2-mediated anti-oxidant enzymes and can regulate blood pressure and lipid profile. Also, neem inhibits vascular smooth muscle contraction through the block of calcium channels and decreases high blood pressure. Neem up-regulates eNOS expression as a vasodilator and increases NO level. Moreover, neem reduces vasoconstriction through the regulation of the ERK1/2 signaling pathway. In the diabetic condition, neem up-regulates GLUT4 and reduces the glucose uptake. Neem also inhibits intestinal enzymes such as glucosidases. Understanding the signaling pathways help to expand the use of neem in the treatment of the MetS. However, few studies have been conducted to investigate the anti-diabetic, anti-hypertension, anti-hyperlipidemia, and anti-obesity activities of neem in humans. Therefore, further clinical studies are needed to assess the protective effects of neem.
